# Sex as a biological variable in ageing: insights and perspectives on the molecular and cellular hallmarks

**DOI:** 10.1098/rsob.240177

**Published:** 2024-10-30

**Authors:** José Héctor Gibrán Fritz García, Claudia Isabelle Keller Valsecchi, M. Felicia Basilicata

**Affiliations:** ^1^ Institute of Molecular Biology (IMB), Mainz, Germany; ^2^ University Medical Center (UMC), Mainz, Germany

**Keywords:** sex chromosomes, sex hormones, hallmarks of ageing, ageing, X chromosome, X-chromosome inactivation

## Abstract

Sex-specific differences in lifespan and ageing are observed in various species. In humans, women generally live longer but are frailer and suffer from different age-related diseases compared to men. The hallmarks of ageing, such as genomic instability, telomere attrition or loss of proteostasis, exhibit sex-specific patterns. Sex chromosomes and sex hormones, as well as the epigenetic regulation of the inactive X chromosome, have been shown to affect lifespan and age-related diseases. Here we review the current knowledge on the biological basis of sex-biased ageing. While our review is focused on humans, we also discuss examples of model organisms such as the mouse, fruit fly or the killifish. Understanding these molecular differences is crucial as the elderly population is expected to double worldwide by 2050, making sex-specific approaches in the diagnosis, treatment, therapeutic development and prevention of age-related diseases a pressing need.

## 1. Introduction

It has become increasingly evident that males and females across species show differences in lifespan (see Glossary) and ageing. In many mammals, females live longer than males, while in birds, males are the longer-lived sex [1]. In humans, women live on average 5 years longer than men [2]. Paradoxically, women are frailer (see Glossary) later in life (usually after the onset of menopause) and do not necessarily have a longer healthspan [3] (see Glossary). Age-associated illnesses are often sex-biased. For example, in 2018, men older than 65 years showed higher death rates of cancer, heart disease, stroke and diabetes, while Alzheimer’s disease (AD), influenza and pneumonia showed higher death rates in women [4]. Women are also more susceptible to autoimmunity [5]. Such age-associated sex biases deserve more attention, as the elderly population worldwide is expected to double from 12% to 22% between 2015 and 2050.

Sex is often not reported in the scientific literature, making it hard to link sex-specific effects to ageing, although sex-biased gene expression is widespread in human tissues [6]. With exceptions, most genome-wide association studies (GWAS) omit sex chromosomes [7]. Lastly, the inclusion of women in clinical trials has been historically limited [8], and their underrepresentation has only recently initiated a callout to understand women’s health across their lifespan [9]. The inclusion of women in clinical trials will avoid restricting the development and the application of current therapies, especially when a female-biased disease is encountered [10].

One reason why either sex can be more prone to certain age-associated phenotypes are the sex chromosomes. In mammals, females are XX and males XY, where the presence of the Y chromosome triggers the development of male gonads and secondary sexual traits. The type of sex determination system, whether it involves heterogametic XY males (see Glossary) as in mammals or heterogametic ZW females (see Glossary) as in birds, has been correlated with adult sex ratios (see Glossary) [1,11]. Mammals share evolutionarily conserved sex chromosomes, but in wild populations, not all mammalian females live longer than males. However, when they do, they have a longer median lifespan of around 20% [12]. How the sex chromosome complement impacts longevity is an active area of research [13,14]. Female mammals undergo X-chromosome inactivation (XCI), in which one of the two X chromosomes becomes heterochromatic, leading to the silencing of most of its genes (see box 1). Recently, genetic and epigenetic changes affecting the inactive X of females as well as the Y chromosome of males have been linked to the development of age-associated traits and diseases.

Box 1. **The X chromosome and X-chromosome inactivation.**

In mammals, sex is determined by the sex chromosomes, with males containing a gene-rich X chromosome and a degenerated, gene-poor Y chromosome; females instead harbour two X chromosomes. *Sry*/*SRY* is the Y-chromosomal male-determining gene, and its expression during embryonic development triggers a signalling cascade for the development of testis from undifferentiated gonads while suppressing ovarian differentiation. An XX genotype results in the expression of genes involved in the development of ovaries [15]. Although the X and Y chromosomes are non-homologous, a handful of X chromosome genes are also present in the Y chromosome. These X–Y pairs are known as gametologues and are mainly located in the pseudoautosomal region. The Y chromosome contains around 60 protein-coding genes of which 17 are gametologues (see Glossary) in humans and 9 in the mouse [16].

The XX versus XY genotype results in a gene dosage imbalance between the sexes with males exhibiting X-linked monoallelic expression. This may disrupt protein stoichiometric ratios and perturb molecular networks [17]. To equilibrate gene expression levels between the sexes, during the embryonic development of female mammals, one X chromosome is chosen for the collective silencing of all its genes. This is referred to as XCI, as reviewed by Galupa & Heard [18].

XCI begins with the upregulation of the master regulator *Xist/XIST* (which stands for X-inactive specific transcript), a long non-coding RNA transcribed from the future inactive X, coating it in *cis* (see Glossary). *Xist* then spreads to the rest of the X chromosome and induces a cascade of epigenetic mechanisms, including histone modifications, macroH2A deposition and DNA methylation. This promotes silencing, late-replication and structural reorganization of the X chromosome. The choice of which X to silence is random, but once a decision is made, the inactivated chromosome is propagated mitotically during each cell division.

XCI does not silence all genes as some are able to escape silencing. The mammalian X chromosome contains roughly a thousand of protein coding genes, and the number of escape genes is estimated to be around 20% in humans [19] and 3–8% in mice [20]. Escape from XCI occurs in a highly tissue-specific and, in humans, also in a variable fashion [18,21–23]. Ten human gametologues and four in mice escape inactivation [24]. Despite their high degree of sequence similarity, gametologues often exhibit functional differences between their X and Y versions [25]. These differences can contribute to sex-biased responses in various biological processes [26,27].

Besides the X and Y chromosomes, the action of sex-specific hormones can also contribute to an increase or reduction in the lifespan of one sex [13,28]. Experimental models such as the four core genotype (FCG) mouse model allow researchers to discern the contribution of gonadal sex versus sex chromosomes to biological processes such as ageing (see box 2).

Box 2. **The FCG mouse model and ageing studies.**

Since sex chromosomes trigger the development of the gonads (XX females develop ovaries, XY males testes), a sex-specific phenotype cannot be unambiguously ascribed to sex hormones secreted by gonads or the cellular karyotype. For this reason, the FCG mouse model was developed. Male gonads in mammals develop due to the presence of a single gene, *Sry*. While *Sry* is found in the Y chromosome, it can induce maleness independently of its chromosomal location. The FCG mouse model makes use of this property, as a transgenic male expressing *Sry* from chromosome 3 develops male gonads independently of the sex chromosome karyotype. Mating of such a male XY^−^ (*Sry*^+^) mouse, in which *Sry* is transferred to an autosome, with normal XX females results in four offspring genotypes or phenotypes: (i) gonadal males with XX (*Sry*^+^) genotype; (ii) gonadal males with XY^−^ (*Sry*^+^) genotype; (iii) gonadal females with XX genotype; and (iv) gonadal females with XY^−^ genotype.

Comparing mice with different gonads, but the same sex chromosome complement, allows researchers to discern the action of sex hormones produced by testis and ovary. On the other hand, sex chromosome complement can be evaluated by comparing mice with the same gonads (XX versus XY) [29]. If in this comparison a difference is detected, other mouse models can help to discern whether those arise from the second X or Y chromosome [30]. Of note, a recent study identified an X-to-Y copy translocation in the FCG model, which impacts the dosage of nine genes [31]. One of these is *Tlr7*, whose excessive dosage leads to autoimmunity. Nonetheless, the FCG model has been a powerful tool to delineate the contribution of gonadal hormones as well as the identification of genes in sex chromosomes that play roles in physiology and disease (for a complete list, see [32]). Not many studies have investigated ageing with the FCG model, but it appears that XX mice, regardless of the gonads, live longer than their XY counterparts and that the presence of ovaries lengthens lifespan only in XX mice [33]. In addition, the extra X chromosome confers resilience to age-dependent cognitive decline [34].

Here we review sex-specific differences in the context of ageing and age-related diseases. Those could arise from social, environmental (not explored in this review) and physiological differences between men and women. We discuss these biological characteristics in the context of the hallmarks of ageing, which are an integrated framework to categorize causes and features of age-associated decline [35,36]. We then focus on how the epigenome of the inactive X and its master regulator *Xist*/*XIST* could contribute to both disease manifestation and resilience.

## 2. Sex bias and the hallmarks of ageing

**Figure 1.**
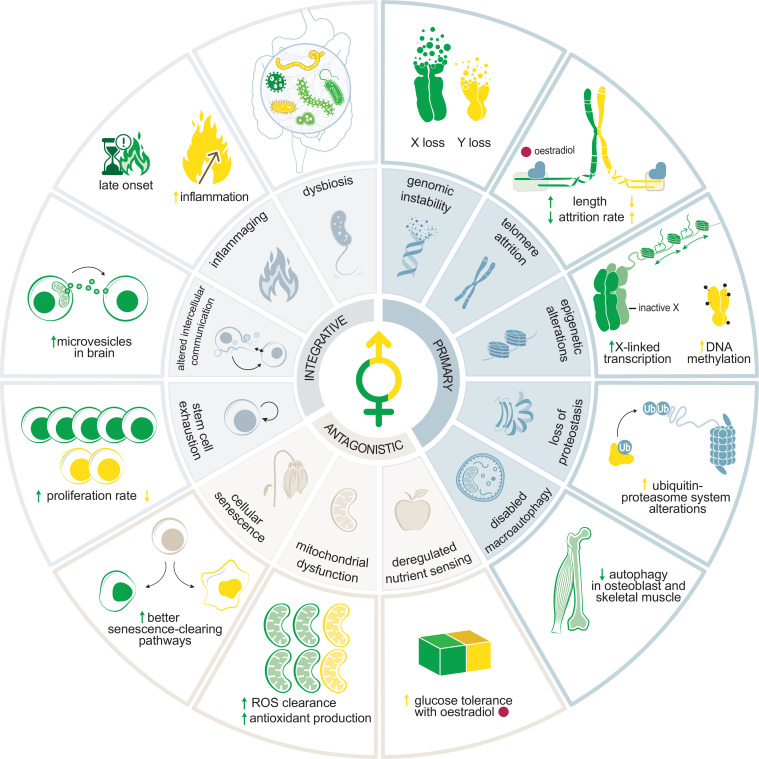
Graphical illustration of the hallmarks of ageing and how they are affected in a sex-specific manner. One example per hallmark (green for females and yellow for males) is illustrated. Primary hallmarks are shaded in light blue. Chromosome instability results in Y chromosome loss in men and X chromosome loss in females. Telomere attrition is faster in males than females, in part due to the oestrogen responsiveness of the TERT subunit of the telomerase complex. Epigenetic alterations include hypermethylation of the Y chromosome and relaxation of the heterochromatic inactive X that leads to the upregulation of X-linked genes. Loss of proteostasis affects primarily males, where X-linked mutations reduce lifespan and influence disease onset. Disabled macroautophagy is female-biased with lower activity seen in osteoblasts and skeletal muscle. Antagonistic and integrative hallmarks are shaded in beige and light grey, respectively. Cellular senescence negatively affects males before females. Mitochondrial dysfunction affects males before females and is associated with impaired clearance of reactive oxygen species (ROS) and production of antioxidants. Nutrient sensing: sex effects depend on the modulation of each pathway. The example shows oestradiol, which improves glucose tolerance in males. Integrative hallmarks: Loss of stem cell potential is faster in males. Altered intercellular communication is illustrated by the increase in mitochondrial-derived microvesicles in female aged astrocytes. Inflammaging affects males more than females. Dysbiosis starts later in females, as menopause impacts microbial diversity.

Ageing is the progressive deterioration of cellular functions over time, compromising an organism’s integrity [35,36]. The proposed hallmarks of ageing comprise cellular and molecular features that contribute to the phenotypic characteristics of the ageing process. They are grouped into three categories and are tightly related to one another [35,36]. The *primary hallmarks* (genome instability, epigenetic alterations, telomere shortening, loss of proteostasis and disrupted autophagy) directly contribute to a decline in cellular function by different means. These are initially counteracted by the *antagonistic hallmarks*, which, over time, become detrimental (cellular senescence, mitochondrial dysfunction and deregulated nutrient sensing). Ultimately, mishandled damage to nucleic acids or proteins leads to stem cell exhaustion, altered cellular communication, chronic inflammation and dysbiosis (see Glossary) (grouped as the *integrative hallmarks*). Sex differences in the hallmarks of ageing have been reviewed by Hägg & Jylhävä [28]. Here, we provide a brief discussion of sex bias in ageing hallmarks (see figure 1), especially in the light of novel insights gained in the last 3 years in humans and other species. When provided, we also report a list of genes that have been correlated with the hallmarks of ageing in a sex-specific fashion (see table 1).

**Table 1.** Genes or loci linked to the ageing hallmarks in a sex-dependent manner in different species. When a locus is reported, we additionally provide the variant or probe used (for GWAS and EWAS). The LOY-related loci identified by Wright *et al*. [37] were also found to be involved in LOX. However the LOX variants had little correlation to LOY in Liu *et al*. [38].

Phenomenon	Species	Bias	Gene/Locus	Location	Ref
**Genomic instability**					
Decreased DNA damage repair efficiency resulting in Fanconi anemia	*Homo sapiens*	Male	*FANCB*	Chr X	[39]
Decreased DNA damage repair via non-homologous end joining in blood lymphocytes	*H. sapiens*	Female	*ATM*	Chr 11	[40]
	*H. sapiens*	Female	*BLM*	Chr 15	
	*H. sapiens*	Female	*XRCC6*	Chr 22	
LOY	*H. sapiens*	Male	*PMF1, PMF1-BGLAP; rs2736609, rs2842873*	Chr 1	[37,41]
	*H. sapiens*	Male	*TSC22D2, LINC01214; rs59633341, rs4681200*	Chr 3	
	*H. sapiens*	Male	*SENP7; rs4683900* *SPINK8, FBXW12; rs115854006* *LINC01214; rs4681200* *FAM172BP,TRMT10C; rs13088318*	Chr 3	
	*H. sapiens*	Male	*NREP, STARD4-AS1; rs56084922, rs56116444*	Chr 5	
	*H. sapiens*	Male	*CCDC162P; rs13191948* *CD164; rs11251* *QKI; rs381500* *MEAT6; rs4709819*	Chr 6	
	*H. sapiens*	Male	*MAD1L1; rs4721217, rs4721217*	Chr 7	
	*H. sapiens*	Male	*RBPMS; rs35091702, rs2979469*	Chr 8	
	*H. sapiens*	Male	*NPAT; rs4754301* *C11orf65; rs227079*	Chr 11	
	*H. sapiens*	Male	*WBP4; rs10687116*	Chr 13	
	*H. sapiens*	Male	*TCL1A, TUNAR; rs1122138, rs1122138* *DLK1, LINC00523; rs137952017, rs72698721*	Chr 14	
	*H. sapiens*	Male	*CENPN, ATMIN; rs12448368, rs77874075*	Chr 16	
	*H. sapiens*	Male	*TP53; rs78378222, rs201753350* *FAM117A; rs77522818, rs78997619*	Chr 17	
	*H. sapiens*	Male	*LINC01478; rs11082396, rs80277818* *BCLN2; rs17758695*	Chr 18	
	*H. sapiens*	Male	*TPX2; rs60084722*	Chr 20	
LOX	*H. sapiens*	Female	*PHC2; rs10798950*	Chr 1	[38,42]
	*H. sapiens*	Female	*S1PR1; rs11166573* *LOC100506274; rs16866241* *CPS1; rs66826907* *SP140L; rs725201, rs17327417, rs3731723, rs3086612, rs11885965, rs184226567, rs11686798, rs1678185*	Chr 2	
	*H. sapiens*	Female	*EOMES; rs2887944* *CX3CR1; rs3732378* *LPP-AS1; rs13080752*	Chr 3	
	*H. sapiens*	Female	*TADA2B; rs568868093* *CENPU; 4:184696883:C:CT*	Chr 4	
	*H. sapiens*	Female	*ERAP2; rs3832368*	Chr 5	
	*H. sapiens*	Female	*JARID2; rs794791* *BTN3A2; rs57760309, rs150289512* *HLA; rs141806003, 6:29866672:A:G* *HSPA1A; rs9266234, rs9267283, rs2844455* *C2; rs115378818* *HLA-DQA1; rs7771548, rs146406015* *ITPR3; rs7752348* *CENPQ; rs9395493* *SCML4; rs1739873* *CENPW; rs9372840* *MYB; rs4895441* *SHPRH; rs148401398* *LOC102724152; rs381500*	Chr 6	
	*H. sapiens*	Female	*MAD1L1; rs2280548*	Chr 7	
	*H. sapiens*	Female	*CSMD1; rs1827666* *MSC; rs10099390*	Chr 8	
	*H. sapiens*	Female	*TNFSF8; rs36118932*	Chr 9	
	*H. sapiens*	Female	*ATM; rs751343*	Chr 11	
	*H. sapiens*	Female	*KLRB1; rs5796352*	Chr 12	
	*H. sapiens*	Female	*CSNK1A1L; rs11147640*	Chr 13	
	*H. sapiens*	Female	*IL27; rs181206* *HEATR3; rs754391850*	Chr 16	
	*H. sapiens*	Female	*TP53; rs78378222* *PRKAR1A; rs768326149, rs16973034*	Chr 17	
	*H. sapiens*	Female	*TOMM40; rs113106418* *LILRA1; rs10411397*	Chr 19	
	*H. sapiens*	Female	*NCOA6; rs2076668*	Chr 20	
	*H. sapiens*	Female	*GSPT2; 23:51749114:C:CGT* *KLF8; rs6521410, rs141849992* *AMER1; rs181043195* *AR; rs58638231* *PLS3; rs12836051, rs60523627* *DXZ1; rs2942875* *DXZ4; rs11091036*	Chr X	
**Telomere attrition**					
Faster telomere attrition rate resulting in dyskeratosis congenita	*H. sapiens*	Male	*DKC1*	Chr X	[43]
**Epigenetic alterations**					
Hypomethyation in muscle, tail, and kidney correlates with decreased expression of androgen receptors	*Ovis aries, Mus musculus*	Male	*MKLN1; cg21524116*	Chr 6 (*M. mus*)	[44]
Hypermethylation in the blood	*H. sapiens*	Male	*NLGN4Y; cg03055837, cg04691144, cg27443332, cg03706273* *LOC100101121, TTTY23; cg00311963* *DDX3Y; cg14180491* *TBL1Y; cg01707559* *TTTY20; cg06636270* *TMSB4Y; cg26198148* *TTTY14; cg03244189, cg13845521, cg11816202, cg15345074*	Chr Y	[45,46]
Hypomethylation in the blood	*H. sapiens*	Male	*TTTY15; cg25032547* *DDX3Y; cg17816615* *E1F1AY; cg01988452, cg13308744* *TTTY13; cg14467015*	Chr Y	
	*H. sapiens*	Female	*GAGE10; cg15833111*	Chr X	[47]
Age-dependent loss in HSCs results in increase X-linked expression, hypomethylation and higher chromatin accesbility	*M. musculus*	Female	*Xist*	Chr X	[48]
**Loss of Proteostasis**					
Decrease or loss-of-function of the E1 ubiquitin-activating enzyme leads to X-linked infantile spinal muscular atrophy and shorter lifespan	*H. sapiens*	Male	*UBA1*	Chr X	[49]
Decrease or loss-of-function of the E2 ubiquitin-conjugating 2A enzyme leads to neurodegenerative diseases	*H. sapiens*	Males if it is a germline pathogenic variant; in ageing, depending on the neurodegenerative disease	*UBE2A*	Chr X	[50]
WT Ubiquilin−2 aggregates in synucleinopathies	*M. musculus, H. sapiens*	No formally tested	*UBQLN2*	Chr X	[51]
Germline mutations lead to ALS and FTD	*H. sapiens*	Earlier onset in males	*UBQLN2*	Chr X	[52]
FMRP mutations lead to adult-onset neurodegenerative disorder, possibly to its role in the ubiquitin pathway	*H. sapiens*	Males	*FMR1*	Chr X	[53,54]
Tau aggregation by increased expression of the USP11 deubiquitinase in the female brain	*M. musculus, H. sapiens*	Females	*USP11*	Chr X	[55]
**Disabled macroautophagy**					
Decreased number of autophagic vesicles in the aged osteoblasts assessed by LC3-II protein levels	*M. musculus*	Female	*Map1lc3b*	Chr 8	[56]
Decreased autophagic activity in the aged osteoblasts is due, in part, to lower number of autophagic vesicles, assessed by LC3-II protein levels	*M. musculus*	Female	*Map1lc3b*	Chr 8	[57]
Pathogenic germline variants can lead to impaired autophagy in the Danon disease	*M. musculus, H. sapiens*	Males are more affected but a later onset is seen in females, resulting in a shorter male lifespan	*LAMP2*	Chr X	[58]
ALS/FTD-related pathogenic UBQLN2 mutations impair ubiquitin-proteasome-driven mitophagy	*H. sapiens*	Earlier onset in males	*UBQLN2*	Chr X	[59]
**Cellular senescence and inflammaging**					
Higher expression in dendritic cells, natural killers, T cells, B cells and macrophages promotes inflammation and oxidative stress	*H. sapiens*	Male	*CXCR4*	Chr 2	[60]
	*H. sapiens*	Male	*HLA-DRB5*	Chr 6	
	*H. sapiens*	Male	*DDIT4*	Chr 10	
	*H. sapiens*	Male	*JUNB, ZFP36*	Chr 19	
Higher expression, related to higher activity of T cells	*H. sapiens*	Females	*CCR4*	Chr 3	
	*H. sapiens*	Females	*CYBA*	Chr 16	
	*H. sapiens*	Females	*IL−2RG*	Chr X	
	*H. sapiens*	Females	*MZB1*	Chr 5	
	*H. sapiens*	Females	*XBP1*	Chr 22	
Decreased inflammation in monoctyes and macrophages	*M. musculus, H. sapiens*	Females	*XIST*	Chr X	[61]
**Dysbiosis**					
KO mice (resembling FXS) have a misregulated gut microbiome	*M. musculus*	No formally tested but FXS is more prevalent in human males	*Fmr1*	Chr X	[62]
Increased expression of these genes is correlated with a stronger immune responses in undisrupted microbiota	*M. musculus*	Females	*Kdm6a, Eif2s3x*	Chr X	[63]
**Stem cell exhaustion**					
Faster attrition rate impairs stem cell proliferation leading to bone marrow failure	*H. sapiens*	Male	*DKC1*	Chr X	[43]
**Altered intercellular communication**					
Higher expression in astrocytes of mitochondria-derived microvesicles	*M. musculus*	Females	*Tsg101*	Chr 7	[64]
	*M. musculus*	Females	*Anxa2, Hspa8*	Chr 9	

ALS, amyotrophic lateral sclerosis; EWAS, epigenome-wide association studies; FTD, frontotemporal dementia; FXS, fragile X syndrome; GWAS, genome-wide association studies; HSCs, haematopoietic stem cells; LOX, loss of the X chromosome; LOY, loss of the Y chromosome.

### 2.1. Genomic instability

Genomic instability is the result of accumulated DNA damage leading to mutations and malfunctions. Cells exhibit various DNA damage repair (DDR) mechanisms and at least some of them act differentially in the sexes and along the ageing process. For example, the repair efficiency of double-strand breaks in peripheral lymphocytes decreases with age in women but not in men [40]. DDR-related proteins have been shown to play sex-specific roles. p53, an autosomal-encoded protein, is a key transcription factor that apart from acting as a tumour suppressor [65] has been shown to regulate the X chromosome status. p53 binds to the murine X inactivation centre and its loss perturbs XCI [66]. Also, the X chromosome encodes p53 regulators such as kinases and deubiquitinases [67]. Since males have just one X chromosome, mutations in those X-encoded p53 regulators can result in a more significant impact in males [68]. This could provide one explanation why age-associated cancer, except for reproductive and thyroid, is male-biased [67,69]. Another relevant DDR factor is the X-encoded *FANCB*. Its pathogenic variants result in accelerated ageing in men [39,70].

Genomic instability can also arise from the expression of transposable elements. The Y chromosome is especially rich in transposons. It has been shown in the fruit fly that a male burden exists when Y heterochromatin loss upon ageing leads to transposon depression [71]. Whether such a ‘toxic Y’ reduces male lifespan and whether this is conserved has been debated, but recent work suggests this is not the case [72].

Genome instability can impact each sex differently if it affects the sex chromosomes directly. Aneuploid cells (see Glossary) can expand through clonal mosaicism (see Glossary), a process taking place in ageing human lymphocytes and other tissues [42,73]. Sex chromosome aneuploidies in ageing occur at a higher frequency than for any autosome, with loss of the Y chromosome (LOY) and X chromosome (LOX) being the major form of somatic mosaicism in males and females, respectively. LOX occurs with a frequency of less than 3% in women below 40 years, increasing to more than 35% in women older than 80 years [38]. It preferentially affects the inactive X [38,74] and is associated with an increased risk of lymphoid leukaemia and acute tonsillitis [75,76], smoking [38], vitamin B complex deficiency [76] and bacterial-caused pneumonia [76]. LOY is found in different cell types [77], increases from around the age of 50 [78–80] and is correlated with a shorter lifespan [81] and the onset of age-related diseases including AD and bladder and prostate cancers [37,41]. The loss of gametologues via LOY [82,83] or loss-of-function mutations [84] could be a relevant determinant of why cancers are usually deadlier and male-biased. Transplantation of Y-lacking haematopoietic stem cells (HSCs) to recipient male mice leads to cardiac malfunctioning and fibrosis as they age [85]. Genes on the inactive X on the other hand can also provide a genetic reservoir for reduced cancer incidence in women [86]. Whether sex chromosome aneuploidies are drivers or consequences of sex-specific ageing is not well understood.

The use of study systems other than humans (e.g. rats [87]), single-cell technologies to study *de novo* somatic mutations [88], as well as recently developed tools that allow the induction of defined aneuploidies in mammalian systems [89,90] will open up new routes to understanding if, how, and why sex chromosome loss impacts ageing.

### 2.2. Telomere attrition

Telomeres are repetitive DNA sequences located at the end of each chromosome. They associate with proteins to protect free chromosome ends from fusions and from activating DDR pathways [91]. Telomeres shorten at each cell division, and once they are so short that they no longer bind telomere-binding proteins, cells undergo death or replicative senescence (see Glossary) [92]. Telomere attrition (see Glossary) occurs due to the absence of telomerase, a ribonucleoprotein (RNP) complex that adds nucleotides to the telomeres. Telomerase activity is repressed in most somatic cell types, and thus telomeres are a key factor in how many times a somatic cell in an organism can divide [93].

It is clear that telomeres shorten during ageing across different tissues, but there has been conflicting data on whether sex is an important factor in telomere homeostasis [94–97]. Mutations in telomerase-associated proteins result in male-biased dyskeratosis congenita, characterized by faster telomere attrition rates and therefore shorter telomere length (TL) [43]. Interestingly, the majority of germline pathogenic mutations causing dyskeratosis congenita occur in the X-linked dyskerin gene (*DKC1*), one of the proteins that stabilizes and positions the telomerase complex [43]. The lower incidence of affected females is due to skewed XCI (see Glossary) [98]. While skewed XCI is tissue-dependent and increases with female age, it is a rare event in newborns and extreme skewing is mainly seen in the context of X-linked pathogenic diseases [99,100].

Besides important X-encoded proteins for telomere homeostasis, chromosome-specific attrition rates can also contribute to a sex bias in age-related diseases [101]. Telomeres on the inactive X undergo faster attrition rates than those on the active X [102]. Chromosomes bearing shorter telomeres and containing large heterochromatin regions, such as the Y or inactive X, are more easily lost, leading to aneuploidy and/or LOX/LOY [103]. Finally, telomere shortening disrupts XCI maintenance by reducing the deposition of H3K27me3, allowing for the reactivation of genes that under normal XCI dynamics are silenced [104]. Recently, mouse models with human-like telomeres [105] and comparable human somatic expression levels of telomerase [106] have been developed, facilitating mechanistic approaches for studying telomere attrition rates and sex differences in human ageing.

### 2.3. Epigenetic alterations

Although there are multiple uses of the term epigenetics, we herein use it to refer to modifications that alter gene expression across multiple cell divisions without changing the primary DNA sequence. Epigenetic regulators include non-coding RNAs, histones and their associated post-translational modifications and DNA methylation (DNAme). DNAme at cytosine residues is one of the most widely studied epigenetic marks [107]. Interestingly, DNAme appears to be one of the most reliable biomarkers to estimate how physiological conditions (‘biological age’) differ from an individual’s chronological age [108,109]. Mathematical modelling has led to the generation of various ‘epigenetic clocks’ that identify specific methylated cytosine sites correlating with accelerated ageing in males [110]. More recently, ‘new clocks’ have identified evolutionarily conserved sites exhibiting age-related changes across different mammalian species [111]. Some of these methylated sites can become hypomethylated with age, which correlates with higher expression levels of androgen receptors across mammalian species [44]. Also, the Y chromosome becomes hypermethylated with age [45–47]. However, mechanistic experiments that causally connect locus-specific methylation changes with ageing and sex are still lacking.

XCI is another paradigm of epigenetic regulation (see box 1), and, perhaps unsurprisingly, disrupted XCI and misregulation of its master regulator *Xist*/*XIST* have been implicated in sex differences in several ageing hallmarks. Increasing evidence marks the immune system not only as being at the heart of several ageing processes but also as one of the most sensitive systems with regard to disruption of XCI. As such, *Xist*/*XIST* regulation and epigenome remodelling of the X during ageing deserve special attention (see §4).

### 2.4. Loss of proteostasis

Proteostasis refers to the regulation of a balanced proteome through processes such as protein synthesis, folding, localization and degradation [112]. Ageing disrupts proteostasis, leading to increased discrepancies between mRNA and protein abundance [113] and sex-specific alterations in the tissue proteome of mice [114,115]. The brain contains the most long-lived proteins with females exhibiting slower turnover rates of these proteins than males [116]. Although this could be seen as a ‘benefit’ of a slow ageing process, it could be detrimental since spontaneously damaged proteins will be present for longer in females. Of interest, many long-lived proteins in the brain are related to female-biased AD [117].

Mechanistically, such observations could be explained by sex-specific alterations in the protein synthesis machinery. For example, protein levels of the ribosomal large subunit complex are higher in the aged female liver [114]. Inefficient handling of misfolded proteins and protein aggregates may also play a role. Long-lived organisms resist protein damage [118], have an active proteasome [119], exhibit higher expression of chaperones and proteasome subunits [120] and show enhanced mitochondrial detoxification of oxidative species [121]. Proteasome activity varies between males and females in a tissue-specific manner. Higher protein degradation activity can be detected in the murine male kidney, murine female small intestine and spinal cord [122] and human female blood cells [123].

For proteotoxic stress occurring in the endoplasmic reticulum and mitochondria, the unfolded protein response (UPR) serves as a protective mechanism that can differ between males and females (reviewed in the context of age-associated diseases by Wodrich *et al*. [124] and gonadal ageing by Okan *et al*. [125] and Rahmani *et al*. [125,126]). For example, female pancreatic β cells in mice demonstrate greater resilience to endoplasmic reticulum stress (see Glossary) compared to their male counterparts [127]. In nematodes, the gonadal-to-soma axis is required for UPR activation in the mitochondria with XX hermaphrodites (see Glossary) being more responsive than XO males [128].

One explanation for why these processes are sex-biased could be the X-linkage of members of the ubiquitin–proteasome system (UPS; see table 1). Loss-of-function mutations or decreased function either shorten lifespan primarily in men [49] or the disease onset is earlier in men [52]; however, female-biased vulnerabilities exist [55]. Deficiency and misregulation of X-encoded UPS proteins are correlated to neurodegeneration [50,51] and earlier cognitive decline in X-linked disorders [53,54].

Currently, there is no systematic study of how and whether the X chromosomal location of proteostasis pathway genes affects lifespan in the two sexes. This could for example be addressed by investigating the highly conserved UPS and UPR system in different species with different sex chromosome complements.

### 2.5. Disabled macroautophagy

Macroautophagy, the main form of autophagy, is the delivery of cytoplasmic components to the lysosome for their degradation and recycling [129]. This process becomes defective during ageing. Autophagic activity is lower in females than in male fruit flies, promoting disruption of the epithelial gut structure and barrier function [130]. In mammals, basal autophagic activity tends to be lower in murine female skeletal muscle and adipose tissues [56,130]. Age-related changes in autophagic activity have been reported in murine female osteoblasts and skeletal muscle [56,57]. As reviewed in [131], some autophagy-related genes and modulators of the autophagy signalling pathway in the brain are X-encoded in mammals but it is unknown whether their chromosomal location contributes to the lower autophagic activity seen in different female tissues.

Chaperone-mediated autophagy is another form of autophagy that declines during ageing. In this process, some proteins are delivered to the lysosomes by binding to chaperones, instead of being membrane-encapsulated [132]. The X-encoded factor LAMP2A is a central regulator of chaperone-mediated autophagy and is thought to be involved in the age-related decline of this pathway. Indeed, mutations in the *LAMP2* gene lead to Danon disease, in which males are more affected and have a shorter lifespan [58]. Moreover, the X-encoded ubiquilin 2 protein impairs autophagy of the mitochondria when mutated [59]. The enrichment of these X-linked genes as key regulators of such pathways provides a rationale for exploring autophagy in sex-related processes. This is of relevance in age-induced XCI relaxation, which has been previously addressed by [104] and will be further discussed in §4.1.

### 2.6. Cellular senescence and inflammaging

Cellular senescence refers to the permanent arrest of cell division. While senescence can prevent further damage and is therefore initially protective, accumulation of senescent cells is harmful. Male mice exhibit a higher number of senescent cells across their lifespan than females [133], and this can be attributed to more efficient clearing of senescent cells by the female immune system [134]. Accumulation of senescent cells in the human immune system, known as immunosenescence, decreases the capacity of immune cells to clear aberrant cells. This, in turn, appears to promote the production of proinflammatory molecules, such as interleukin 6 and 18, which accumulate prematurely in ageing men [135]. Over time, the constant state of low-grade chronic inflammation is known as inflammaging [136]. Inflammaging has tissue-specific outcomes and is, for example, greater in the female cortex of the ageing brain [137,138]. Fibrosis and inflammation also play a major role in the ageing of the female reproductive tract [139], a property that is modulated by cycling and pregnancy numbers.

Ageing amplifies the sex differences in immune cell types found in young individuals and affects gene expression programmes in a sex-specific manner. For instance, while monocyte numbers do not change between the sexes during ageing, their chromatin accessibility and gene expression are higher in males [60,135]. Another mechanism that shows sex specificity is attributed to the X chromosome. Upon inflammation in monocytes and macrophages, *Xist*/*XIST* surprisingly shows a cytoplasmic localization. Here, this long non-coding RNA interacts with p65 in order to dampen the proinflammatory NF-κB complex [61]. Many questions are left unanswered including how *Xist*/*XIST* senses inflammation and how this nuclear RNA can translocate to the cytoplasm. More studies are needed to uncover whether *Xist*/*XIST’s* role in inflammation diseases is more general [140].

### 2.7. Mitochondrial dysfunction

Mitochondria and the metabolites they produce are a central focus of ageing research, as mitochondrial activity declines over the lifespan [36]. While reduced energy production in ageing can help limit reactive oxygen species (ROS; see Glossary) as their levels decrease too, ROS clearance pathways are also impaired, thus leading to higher oxidative stress. Mitochondrial numbers decrease upon ageing, with lower numbers observed in men [141]. Females exhibit refined mitochondrial processes that can contribute to their lifespan lengthening while delaying disease. Those include higher energy production, higher content of antioxidants to clear ROS and better responses to mitochondrial-induced stress [142]. This can be, for example, observed in the sexually dimorphic bioenergetic profiles of immune cells [143,144] or expression changes in genes important for mitochondrial function in the heart [145]. The FCG model (see box 2) revealed that the Y chromosome, independently of the sex hormones, can be responsible for upregulating *Hk2* and *Pdk4*, both of which are nuclear-encoded mitochondrial genes [146]. However, if this is relevant to sex-specific differences in ageing remains to be analysed.

The mitochondria’s crucial role in energy-demanding tissues is underscored by mitochondrial DNA (mtDNA) mutations (e.g. induced by ROS) occurring in ageing. How mtDNA mutational load affects the sexes needs further investigation, but interestingly, those processes have been associated with the property of mutated mitochondria to become ‘selfish’ and negatively affect the fitness of the ageing host [147]. Of note, mitochondria are maternally inherited, and assumptions that female-specific processes have evolved to maintain mitochondrial function and mtDNA have been proposed [148]. Thus, the question of whether and how mitochondrial haplotypes selected in females contribute to sex-specific ageing will be an intriguing area of study.

### 2.8. Deregulated nutrient sensing

As our bodies age, they handle nutrient sensing, uptake and metabolism in increasingly complex ways. Dietary restriction can extend lifespan in some animals, but its effectiveness diminishes with age [149]. Inhibition of the kinase mammalian target of rapamycin (mTOR) pathway by rapamycin acts as one of the major nutrient-sensing pathways. By inhibiting mTOR, rapamycin mimics caloric restriction and increases lifespan from yeast to worms to mice. Interestingly, it interacts with sex in different ways. In the fasted state, the mTOR pathway generally exhibits higher basal activity in females than in males [150]. In Drosophila, the activation of the mTOR/S6K enhances inflammation through atypical NF-κB activation in fat cells, while its inhibition extends female lifespan. This is not through the downstream autophagic flux, which responds similarly between the sexes [151]. One of the major downstream targets regulated by mTOR is adenosine monophosphate-activated protein kinase (AMPK), which is activated in response to low cellular energy levels. In the killifish, constant AMPK activation appears to benefit females. The constant genetic activation of the AMPKγ1 isoform generates a youth-like response to fed-fasted feeding switches in the adipose tissue of old females [152]. Conversely, when AMPK is activated through direct inhibition of the nucleotide salvage pathway, this extends lifespan in males through improved liver function [153]. In mice, interfering with AMPK produces mixed results with regards to which sex profits from the interventions, potentially due to variations in genetic background and the specific tissues analysed [150]. Since the evolutionary origins of the X and Y chromosomes in killifish, fruit fly and mammals, and accordingly, their gene compositions are entirely different [154], it is unlikely that sex-biased effects on mTOR arise from X-linkage.

Indeed, sex hormones influence how the body interprets nutrient signals. For example, oestradiol (see Glossary), the female sex hormone, improves glucose tolerance in males but not females [155]. It would be interesting to understand the molecular basis of these sex-specific differences in nutrient sensing, but because hormones, X chromosomes and dietary requirements differ for various model organisms, it will likely be challenging to extrapolate the findings to humans.

### 2.9. Dysbiosis

The commensal bacteria found in the mammalian gastrointestinal tract are important for nutrient absorption and digestion, protection against pathogens and synthesis of beneficial metabolites. The composition and function of the intestinal microbiome change considerably during ageing, which in turn influences its interaction with the immune system and thus correlates with e.g. increased susceptibility to infectious diseases and reduced vaccination response.

Compared to males, the female gut microbiome is more diverse [156] and harbours, for example, species that ensure better glucose metabolism [157]. This greater abundance of beneficial bacteria in the female gut is kept until menopause [158]. After menopause, the female gut microbiome becomes similar to men [159]. Depletion of the gut microbiome in mice significantly alters sexually dimorphic gene expression, particularly in the liver. Germ-free male mice show downregulation of male-biased genes and upregulation of female-biased genes, while germ-free female mice exhibit attenuated female-biased gene expression. Those genes belong to metabolic pathways regulated by sex hormones and growth hormonal signalling. The gut microbiome thus appears to be crucial to maintaining proper sexual differentiation of gene expression and metabolism in mice [160]. The transit time (see Glossary) is also associated with the gut microbiome and varies between the sexes [161].

Sex-dependent changes in microbial diversity can, for example, influence cytokine (see Glossary) production [162]. Since microbial diversity is known to be altered in, e.g. Fragile X syndrome [62], genes encoded by sex chromosomes may be causally related to sex-dependent dysbiosis upon ageing. In a recent study, this idea was investigated using the FCG mouse model. XX mice showed a better immune response to dead bacteria than XY mice, regardless of gonadal sex. Although clearance of the gut microbiome by administration of antibiotics impaired this effect, it was restored after recolonization of the intestine with bacteria that produce short-chain fatty acids, a known class of immunomodulators. At the molecular level, these effects were associated with expression changes in the X-linked genes *Kdm6a* and *Eif2s3x*, which both belong to the class of XCI escapee [63]. Some X chromosome escape genes have been linked to promoting acute proinflammatory cellular responses, as observed in sex-biased human glioblastoma [86]. Alternatively, Y-linked gametologues could positively influence the immune response in XY individuals. For example, functional divergence has been demonstrated to occur in the context of the Uty paralogue of Kdm6a through a protective activity against pulmonary hypertension [26,27,163]. These hypotheses are yet to be tested in the context of the gut-microbiota axis.

### 2.10. Stem cell exhaustion

Tissue-residing stem cells are important to renew tissues such as the gut, skin or blood. Their function declines with age and many stem cell populations face decreased potential during ageing [164,165]. HSCs are the go-to cell type for tissue stem cell biology as their derived mature cells (e.g. erythrocytes or macrophages) are largely short-lived and cannot proliferate. Ageing results in decreased HSC potential [166], cessation of proliferation in up to 30% of its population [167] and HSC size increase, which is negatively associated with functionality [168]. Cell cycle analysis indicates that aged HSCs are in a more quiescent state than young ones [169]. Given that proliferation is central to stem cell biology, it is unsurprising that the functional decline of stem cells can be linked to primary hallmarks, such as DNA damage [170] and telomere attrition. For instance, bone marrow failure is the main cause of death in dyskeratosis congenita [43].

How is stem cell function different between males and females and how does it impact ageing? The adult intestinal stem cells of the fruit fly show intrinsic sex differences reflecting on the expression of genes involved in growth and metabolism [171–173] impacting the cell cycle and thus susceptibility to cancer [68]. Differences associated with the cell cycle are also observed in rodents, where haematopoietic, neural and muscle stem cells proliferate faster in females [174]. It will be important to study and analyse sexes separately in the recently developed stem cell tracing single-cell gene expression technologies (e.g. [167] used both male and female individuals together). Different stem cell-containing tissues need to be studied and ideally involve comparisons of humans to other systems whenever possible.

### 2.11. Altered intercellular communication

Cell–cell interactions can impact ageing by activating inflammatory pathways leading to enhanced immunosenescence [60]. Yet, there has been little research into sex-biased changes in intercellular communication with ageing. One observation relates to cell–cell communication occurring through extracellular vesicles. These vesicles are secreted by cells and transfer different macromolecules to others [175]. Through increased levels of biogenesis factors (e.g. Annexin 2, Alix, TSG101 and HSC70), microvesicle numbers increase upon ageing in female but not male mouse brains. These vesicles are of mitochondrial origin and are produced by astrocytes [64]. The content of these vesicles, the target cells, as well as the molecular nature of the sex bias, remains obscure. Extracellular vesicles have also been correlated with female-dependent neuroinflammation in ageing [176]. Finally, other forms of perturbed intercellular communication have been reported between (e.g. granulosa cells and the ageing oocyte [177]).

## 3. The roles of sex hormones in human ageing

The major sex hormones are testosterone and oestradiol, which are important for the development of sexual organs and traits. Both are present in males and females but in different amounts. Testosterone is the major male sex hormone and oestradiol—metabolized from testosterone by the enzyme aromatase—is the major female sex hormone. Humans experience a ‘mini-puberty’ (see Glossary) in early infancy, and both hormones are detected circulating in the human body at low levels. However, their main peaks start during puberty between 8 and 13 years in girls and 9 and 14 years in boys. In the mouse, this corresponds to 4–6 weeks postpartum in females and 7–11 weeks in males [178,179]. In men, testosterone decreases steadily from around the age of 30 years and is accompanied by an increase in oestradiol levels. Eventually, this can result in a state referred to as andropause and is characterized by fatigue, insomnia, mood changes, irritability and lower sexual desire. Between 40 and 49 years, the frequency of andropause among men is 0.1%, increasing to 5.1% by 79 years [180]. Oestradiol in females is dynamic with constant cycling during menstruation after puberty. Between 35 and 45 years of age, females enter perimenopause, characterized by irregular hormonal changes. After this—post-menopause—oestradiol levels drop abruptly [181].

Sex hormones exert their effects by binding to membrane-bound or intracellular receptors, which are expressed in a variety of cell types, tissues and even organelles [182,183]. They regulate brain sexual differentiation [184], change chromosomal three-dimensional conformation [185] and induce sex-biased expression profiles, to some extent through hormone-related transcription factors [6]. The roles of sex hormones in healthspan and lifespan are actively investigated (comprehensively reviewed by Ng & Hazrati [186]). Nonetheless, it appears that sex hormones are protective, a property from which both sexes can ‘profit’. For instance, premature menopause is associated with a shorter lifespan and higher mortality rates [187]. In mouse skeletal muscle cells, oestradiol is present in mitochondria, reducing oxidative stress and increasing mitochondrial respiration [188]. In males, the adipose tissue is an inflammatory hub, and testosterone alleviates this by reducing fat mass [189].

If both sex hormones hold anti-ageing properties, why do men age faster? The decline in testosterone in men starts earlier than the oestradiol decline in women. Such a decline in males does not trigger specific diseases but rather increases the risk of developing them. For example, in men with andropause, there is a co-occurrence with diabetes, osteoporosis and increased fat mass [190]. In females, many of the hallmarks of ageing worsen during peri- and post-menopause. For instance, levels of the antioxidant glutathione are higher in the female brain during the reproductive age, offering protection against ROS. Glutathione decline leads to the remodelling of metabolic pathways that expose females to mitochondrial dysfunction [191]. Possibly, oestradiol could be also more protective than testosterone. However, the oestradiol pulses during the reproductive age of mice come at a price as the constant remodelling of the female reproductive organs in each cycle results in age-dependent fibrosis and inflammation [139].

The supplementation of sex hormones, also referred to as hormonal therapy (HT; see Glossary), is an opportunity for ameliorating ageing-induced symptomatology. This is of particular interest in females given the sudden oestradiol decrease, but can also be applied to men [192,193]. Ideally, HT is provided early enough so that its protective effects can be sufficiently sustained, usually before 60 years [194]. The cellular response upon HT is cell-type-specific. Oestrogen therapy in post-menopausal women reduces the production of proinflammatory cytokines [195] while increasing B cell counts [196]. In mice, the mammary tissue responds rather negatively to exogenous oestrogen by increasing the risk of developing breast cancer [197,198]. Hormonal phases influence the onset and incidence of colorectal cancer, with oestrogen supplementation being associated with a lower risk of developing the disease in post-menopausal women [199,200]. More research is needed to understand how different human cell types respond to age-related HT, including testosterone effects. An example includes the development of a hormonal signalling map as it has been recently done in the mouse lemur [201]. While animal models offer insights, establishing clear parallels with human responses is crucial for developing more effective, personalized HTs while reducing side effects.

Additionally, many issues still need to be addressed and properly designed case-control studies with the appropriate control subjects are needed. For instance, many females undergo hormonal changes when they use contraceptives for birth control [202]. How they affect female lifespan is not clear, but combinatorial therapies decrease the risk of developing colorectal, endometrial, ovarian, lymphatic and haematopoietic cancer, with the effects persisting for at least 30 years in past users [203]. How the constant use of hormones can modify male healthspan, including the risk of cardiovascular disease and death, is less well studied, but bodybuilders, who consume androgens as part of their activities, are an ideal case study to do so [204]. Further investigations are also needed to determine if these beneficial effects persist with the newer generation of birth control pills. When needed, the use of emerging organisms in ageing research can provide deeper mechanistic insights into these processes [205].

## 4. Balancing the seX: opposing effects of the X chromosome

**Figure 2.**
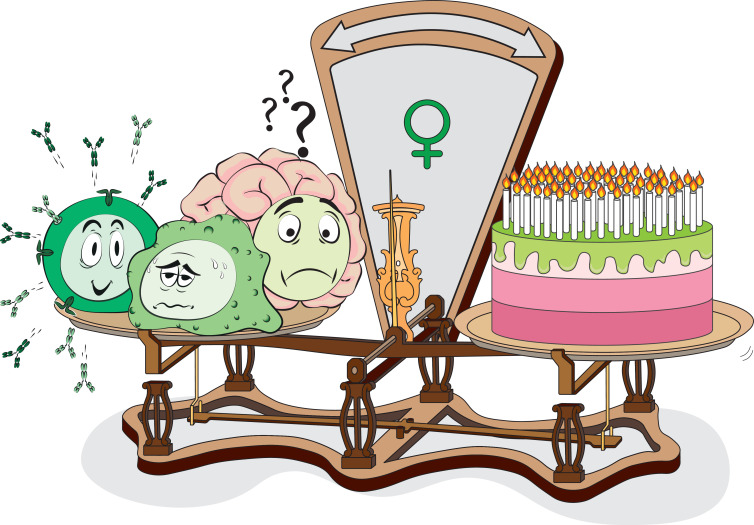
Artistic illustration of the opposing effects of the double X chromosome dosage in females. The biallelic expression of some genes on the second (and inactive) X chromosome in female mammals extends their lifespan and consequently, females celebrate more birthdays (right-hand plate). While their lifespan is longer, having that additional X chromosome comes at the expense of a worsened healthspan (frailty), and thus having a higher risk of autoimmunity and specific neurological disorders (left-hand plate).

XCI (see box 1) provides expression equilibration of X-linked genes between the sexes but can also benefit females. Although XCI is usually random, it can become skewed to one of the parental chromosomes, which is relevant when genes are mutated in a heterozygous fashion. This can prevent the expression of pathogenic variants, a possibility that males do not possess (for a detailed discussion see [206]). XCI can also buffer the effect of cancer mutations occurring on the X [207,208]. Lastly, the biallelic expression of female escapees can provide resilience, for example, if they encode tumour-suppressor genes [84]. The two X chromosomes are beneficial to female lifespan and healthspan, but, nonetheless, can also affect females negatively (see figure 2).

### 4.1. Moonlighting roles of *Xist/XIST* beyond triggering XCI

*Xist*/*XIST* establishes XCI, but once this repressive state is induced, it is dispensable for maintenance, making XCI a *bona fide* epigenetic mechanism [209,210]. Nonetheless, there are exceptions to the rule, with ageing being one process associated with XCI relaxation in some loci of human skin fibroblasts [211] and whole tissue murine spleen and kidney [212]. Cells of the female immune system are especially sensitive to *Xist*/*XIST* loss, resulting in differentiation defects during haematopoiesis and aggressive blood cancer [48,213]. Aged HSCs show perturbed *Xist* localization resulting in hypomethylation, chromatin accessibility changes and increased variability in gene expression [214]. In mature naive lymphocytes, *Xist/XIST* becomes dispersed and only re-associates with the inactive X upon stimulation. Nonetheless, the spatial redistribution does not result in a global re-expression of X-linked genes as one would perhaps expect: female naive lymphocytes show expression profiles highly similar to males, with only few genes being bi-allelically expressed [215,216]. Similarly, in a mouse model with mildly impaired *Xist* function, phenotypes become penetrant in an age-related fashion with escapees being the primary targets [217]. One prominent escape gene and regulator of the innate immune system is *TLR7*. Its expression in *XIST*-depleted B cells leads to the generation of a female-specific B cell population that accumulates with age and can also be observed in autoimmune disorders [218].

Consistent with this, misregulation of *Xist/XIST* RNA is also observed in autoimmunity [219–221]. *Xist* deletion in B cells is sufficient to induce features of autoimmune diseases [222]. Intriguingly, *Xist* may exert female-biased autoimmunity unrelated to its role in XCI. *XIST* alone serves as a ligand to trigger autoimmunity pathways [223]. Moreover, RNPs associated with *Xist*/XIST RNA can serve as autoantigens, which leads to the production of autoantibodies [224]. While autoimmune diseases such as systemic lupus erythematosus or Sjörgen syndrome are rather rare in the elderly population, autoantibodies increase with age, presumably as a result of tissue damage and apoptosis. While strong evidence supports the role of *Xist*/*XIST* in autoimmunity, there are contradictory changes of *Xist/XIST* expression in the brain [225–227], which are yet to be linked to possible age-related neurological illnesses.

In summary, these examples demonstrate that the roles of *Xist*/*XIST* can go beyond canonical XCI. Within its canonical RNPs, *XIST* may regulate autosomal genes [228]. Besides that, it may act as a ligand on its own [224] and inhibit the action of other regulatory factors in compartments other than the nucleus [61]. Since *Xist*/*XIST* is an abundant transcript in females, these possibilities are certainly worthwhile studying in the context of ageing and should encompass tissues other than the immune system.

### 4.2. Two Xs are better than one: escaping provides resilience

How can the X-linkage of genes regulating any of the hallmarks discussed above be responsible for sex bias in ageing? This question becomes striking for the immune system, as 172 immune-related genes are found on the human X (15% out of all X-linked genes), making it the chromosome containing the highest density of such genes [229,230]. The female immune response is more powerful than that of males, and females contain more circulating immune cells, but this renders women more prone to autoimmunity [231]. Their susceptibility appears to arise from the XX karyotype as demonstrated using the FCG model [232].

Besides Tlr7, the X-linked histone demethylases Kdm6a and Kdm5c appear to influence sex-specific ageing by being escape genes [19,20]. Kdm6a escapes XCI leading to sex-biased expression in, e.g. CD4^+^ T cells from mice and humans. Conditional deletion of Kdm6a in CD4^+^ T female cells alleviates neuropathology in multiple sclerosis mouse models by a general decrease in proinflammatory cytokines [233]. XX, compared to XY and XO, mice are more resilient to AD, and this has been correlated with Kdm6a, where overexpression in males attenuates amyloid beta-dependent cognitive decline [234]. More recently, overexpression of catalytically dead Kdm6a in the hippocampus of male mice improved memory and learning upon ageing [235]. Kdm5c is another X-linked demethylase responsible for sex-specific phenotypes. Kdm5c and Kdm6a have been proposed to have a protective role against coronary heart disease in young females [236,237]. However, when a double dose of Kdm5c is expressed in murine adipocytes, this leads to higher weight gain and body fat after a high-fat diet [238], thus highlighting the context-specific benefits and detriments of escape.

Due to the often subtle dosage effects between XX and XY individuals, there is an incomplete understanding of how escape from XCI contributes to sexual dimorphism in age-related diseases. Because escape appears to be highly tissue-specific and can occur in a variable fashion, such studies will be challenging but clearly necessary to advance tailored therapies for men and women.

## 5. Concluding remarks and future perspectives

Sex differences in lifespan and ageing involve a combination of factors including the X chromosome and hormones. Because males and females age differently, it is imperative to investigate the fundamental basis of sexual dimorphism across that process. The inclusion of both sexes in scientific studies, independently of the study system, is essential to understanding how ageing affects each sex (e.g. reviewed by Lushchak *et al*. [239]). A step towards closing this gap is the policy established by the National Institute of Health to consider sex as a biological variable [240]. In addition, exploring sex hormones within the framework of ageing and life history will be fruitful. For example, in Drosophila, sex steroids affect intestinal physiology [241], while pregnancy transiently remodels the female mouse brain [242]. In the killifish, ablation of the germline leads to an enhanced DDR in females, while extending lifespan and improving metabolic functions in males [205]. Pregnancy can also protect against age-related fibrosis and inflammation caused by reproductive cycling in humans [139], thus underscoring its relevance as a biological variable in future studies. Equally important will be to integrate the X and Y chromosomes in GWAS, which will allow for the identification of sex-linked alleles contributing to age-related diseases.

Research using humans is challenging, with a limited number of deeply mechanistic experiments being feasible [243]. Thus, except for a handful of examples (LOY as a driver in uveal cancer [244] and leading to age-related cardiac dysfunction [85]; *Xist* KO leading to autoimmunity [217]), many of the phenotypes observed during ageing (e.g. LOX, changes in methylation loci, *Xist* downregulation and XCI skewing) lack direct evidence of a causative impact on ageing. They could also reflect an adaptation to other processes occurring along the ageing process. Besides further focus on mechanisms, extending the ageing field to samples and organs other than the blood and the haematopoietic system will be also necessary. For instance, the observation of non-canonical regulation of *Xist/XIST* in lymphocytes has been recently recapitulated in mouse and human lung alveolar type 2 cells, which display a high degree of XCI escape [245]. XCI seems apparently more plastic in humans than in mice and sex-specific programmes govern function in many human organs [6].

Ageing is the common functional decline every human faces, and yet we still do not understand how to successfully tackle it. More research is needed in order to provide therapies for healthy ageing that every man and woman can benefit from.

## 6. Glossary

- Adult sex ratio: the proportion of male-to-female individuals in an adult population.
- Aneuploidy: alterations in chromosome numbers that deviate from the euploid complement (e.g., two copies for human autosomes).
- Clonal mosaicism: amplification of cells harboring a different genotype than the inherited germline genome as a result of unrepaired mutations.
- Cytokine: a group of secreted peptides or proteins that have an effect on target cells by typically affecting their growth, proliferation or differentiation.
- *cis*: on the same chromosome where a given gene or regulatory molecule (e.g., *Xist/XIST* non-coding RNA) is encoded.
- Dysbiosis: also known as dysbacteria, refers to an imbalance in the intestinal flora
- Endoplasmic reticulum stress: accumulation of unfolded or misfolded proteins in the endoplasmic reticulum
- Frailty: the condition of vulnerability to worse health outcomes.
- Gametologues: homologous genes between the X and Y chromosomes.
- Healthspan: the length of time an organism lives in good health, free from major limitations.
- Hermaphrodites: organisms harboring male and female gonads.
- Heterogametic sex: the sex of a species with two different sex chromosomes (males in the XY and females in the ZW determination systems).
- Homogametic sex: the sex of a species with two homologous sex chromosomes (females in the XX and males in the ZZ determination systems)
- Hormonal therapy: the use of hormones for medical treatment.
- Lifespan: the period of time for which an organism lives.
- Mini-puberty: activation of the hypothalamic-pituitary-gonadal axis between birth and the first few months of life in humans.
- Oestradiol: main active form of estrogen during the female reproductive time (note that other estrogen-derived molecules exist).
- Reactive oxygen species: highly active chemicals with unpaired electrons arising from cellular metabolism. Examples include superoxide (O_2_^-^), hydrogen peroxide (H_2_O_2_) and nitric oxide (NO).
- Replicative senescence: the phenomenon of an irreversible block to cell proliferation after a cell has completed a defined number of cell cycles (also known as the Hayflick limit).
- Skewed XCI: non-random inactivation of the X chromosome, i.e., when one of the two parental X chromosomes is preferred for inactivation.
- Telomere attrition: time-dependent telomere length shortening.
- Transit time: the time it takes for food to travel through the gut.

## Data Availability

This article has no additional data.
